# Complete Genome Sequences of Classical Swine Fever Virus Strains Isolated from Wild Boars in South Korea

**DOI:** 10.1128/genomeA.00147-13

**Published:** 2013-04-18

**Authors:** Hye-Young Jeoung, Ji-Ae Lim, Seong-in Lim, Jae-Jo Kim, Jae-Young Song, Bang-Hun Hyun, Yong Kwan Kim, Dong-Jun An

**Affiliations:** Animal, Plant and Fisheries Quarantine and Inspection Agency, Anyang, Gyeonggi-do, Republic of Korea

## Abstract

Classical swine fever is a disease that is devastating the pig industry worldwide. Here, we report the complete genome sequences of two classical swine virus strains (YC11WB and PC11WB), isolated from Korean wild boars in 2011. Both strains belong to subgenotype 2.1b. The complete genome sequences of PC11WB and YC11WB are more similar to that of strain ZJ0801 (isolated in China) than to that of the SW03 strain isolated from domestic pigs in South Korea.

## GENOME ANNOUNCEMENT

Classical swine fever virus (CSFV) belongs to the genus *Pestivirus* within the family *Flaviviridae*, which also includes border disease virus (BDV) and bovine viral diarrhea virus 1 (BVDV-1) and 2 (BVDV-2) (1). Classical swine fever (CSF) is a highly contagious disease that can cause heavy economic losses due to its resulting high mortality. Since 2002, there has been a genotype shift from CSFV genotype 3 to CSFV genotype 2 in domestic pigs in South Korea (2). Despite a nationwide program of mandatory vaccinations, sporadic outbreaks caused by CSFV genotype 2 were reported until 2009 (3). However, no outbreaks of CSF have been reported on domestic pig farms in 3 years. Unfortunately, we have isolated two CSFV strains from samples collected from wild boars hunted between 2010 and 2013. Strain YC11WB was isolated from a wild boar hunted in the Yenchon region in May 2011, and strain PC11WB was isolated from a wild boar hunted in the Pochen region in November 2011.

Total RNA was extracted from the blood of wild boar using the microcolumn technique-based QIAamp viral RNA mini kit (Qiagen). cDNA was amplified using a one-step real-time PCR (RT-PCR) kit (Qiagen) using specific primers designed against CSFV genome sequences (4, 5). The RT-PCR amplification products were cloned into the pGEM-T plasmid and sequenced using T7 and SP6 primers and an ABI Prism 3730xi DNA sequencer. The YC11WB and PC11WB genome sequences showed 96.9% homology at the nucleotide (nt) level and 98.5% homology at the amino acid level.

Comparative analysis of the YC11WB Npro, C, Erns, E1, E2, p7, NS3, NS4A, NS4B, NS5A, and NS5B nt sequences with those of a reference strain, SW03 (a genotype 2 virus isolated in South Korea in 2003), revealed high sequence homology: 95.4% for the Npro genes, 94.9% for the C genes, 94.7% for the Erns genes, 94.4% for the E1 genes, 94.5% for the E2 genes, 95.7% for the p7 genes, 95.5% for the NS3 genes, 95.8% for the NS4A genes, 94.0% for the NS4B genes, 95.0% for the NS5A genes, and 95.2% for the NS5B genes. A similar analysis of 75 complete CSFV genome sequences deposited in GenBank revealed that the YC11WB and PC11WB strains showed 96.9% and 97.9% sequence homology, respectively, at the nt level with strain ZJ0801 (accession no. FJ529205). ZJ0801 was isolated in China in 2008.

A phylogenetic tree based on E2 partial (190 nt) sequences derived from 120 CSFV strains deposited in GenBank showed that strains YC11WB and PC11WB belonged to subgenotype 2.1b, whereas CSFV strains isolated from a wild boar in Germany belonged to genotype 2.3 (MEGA 4.1 program) (6).

In summary, the YC11WB and PC11WB strains were isolated from wild boar hunted in neighboring areas in South Korea that are contiguous with the North Korean border. Both strains belong to subgenotype 2.1b, which was responsible for outbreaks in domestic pigs from 2002 to 2009. Knowing the virus strain is very important because it indicates whether it comes from another country or whether it has been transmitted from domestic pigs to wild boar.

### Nucleotide sequence accession numbers. 

The complete genome sequences of the YC11WB and PC11WB strains have been deposited in GenBank under accession no. KC149990 to KC149991.
